# Depression in brain tumor patients—early detection and screening

**DOI:** 10.1007/s00520-023-07785-5

**Published:** 2023-05-16

**Authors:** Alida Finze, Laura Deleanu, Christel Weiss, Miriam Ratliff, Marcel Seiz-Rosenhagen

**Affiliations:** 1 Department of Surgery, University Hospital Mannheim, Medical Faculty of Heidelberg, Theodor-Kutzer-Ufer 1-3, Mannheim, 68167 Germany; 2 Department of Medical Statistics and Biomathematics, Medical Faculty Mannheim, University of Heidelberg, Mannheim, Germany; 3 Department of Neurosurgery, University Hospital Mannheim, Medical Faculty of Heidelberg, Mannheim, Germany; 4 Department of Neurosurgery, Municipal Hospital Memmingen, Memmingen, Germany

**Keywords:** Depression, Glioblastoma, Brain tumor, Neuropsychological screening

## Abstract

**Background:**

Depres sion is reported in up to 90% of cancer patients but to this date, a standardized screening tool for depression specifically modified for patients diagnosed with brain tumors is lacking. Thus, this study aims to develop an adapted screening tool and identify a suitable time slot for screening.

**Methods:**

Sixty-one patients with brain lesions were interviewed prior to neurosurgical resection. For screening purposes, established depression scores were used. A study-specific questionnaire (SSQ) was developed based on patient interviews prior to the trial. Two subgroups were analyzed: patients with benign and patients with malignant tumors (including brain metastases). As a subgroup within malignant lesions, patients with glioblastoma (GBM) were also analyzed separately.

**Results:**

Of patients, 87.5% with GBM presented with results > 16 points on the Center for Epidemiologic Studies Depression Scale (CES-D) after surgery. A decline in patients with benign brain tumors (*p* = 0.0058) and an increase in patients with malignant tumors (*p* = 0.0491) could be shown over time for CES-D scores. In this study, we established a new prototype screening tool for depression. In patients diagnosed with GBM, the number of patients needed to screen for identification of symptoms of depression was 1.59. Best time for screening was 35 days after surgery.

**Conclusions:**

Considering the high prevalence and low number needed to screen of depression in patients diagnosed with GBM, we strongly encourage their routine screening during follow-up appointments (35 days after surgery). We encourage a plan to further establish the questionnaire developed in this pilot study.

**Supplementary Information:**

The online version contains supplementary material available at 10.1007/s00520-023-07785-5.

## Introduction

An average of 86,000 people in the USA were diagnosed with brain tumors annually from 2014 to 2018, 29% of them being malignant brain tumors as reported by the Central Brain Tumor Registry of the United States (CBTRUS) [1].

Previous studies have shown that around 90% of patients diagnosed with malignant brain tumors develop a clinically relevant depression [2]. The prevalence of depression in patients with malignant brain tumors is significantly higher compared to the prevalence in patients diagnosed with cancer overall (15–29%) [3, 4].

In 2007, Giese-Davis et al. reported the impact of depression in patients diagnosed with breast cancer as an independent risk factor for survival. Patients with increasing symptoms of depression presented with a median survival of 25.1 months, while patients with decreasing symptoms of depression had a median survival of 53.6 months [5].

In addition, it is known that depression increases pain perception [6] as well as the risk of cardiac events [7]. Depression also negatively impacts the quality of life [8–10]. In summary, not only is depression a common finding in cancer patients but depression also negatively impacts oncological outcomes. To understand the role of depression in patients with brain tumors in general and with malignant brain tumors in particular better, more evidence is needed.

The aim of this study was to confirm the number of patients at risk for developing symptoms of depression as well as to emphasize the importance of screening in this special patient group. We aimed to create an easy and fast prototype screening tool for the early detection of patients with risk for depression. Furthermore, we aimed to find a practical and sensitive time point for screening. For this, we compared patients with benign brain tumors to patients diagnosed with malignant brain tumors. So far, there is no data on a recommended time point for screening as well as a specific screening tool.

## Material and methods

Data was collected between December 2016 and July 2018 at the University Hospital Mannheim.

Patients aged between 18 and 80 years with a suspected brain tumor based on a MRI (magnetic resonance imaging) or CT (computed tomography) scan were recruited prior to surgery and were included in the study after written consent.

Patients were interviewed prior to surgery (baseline), once between days one to four after surgery and on day 7, 21, and 35 and 6 to 8 weeks after surgery. Patients were asked to fill out the SSQ on the day they received their final histopathological diagnosis and on the following 3 days, in addition.

It was an inclusion prerequisite that the patient has the ability to read and comprehend the German language, draw, and see to an extent that allowed the completion of the following tests:

Beck Depression Inventory II (BDI-II), the German version of the Center for Epidemiologic Studies Depression (CES-D), and a study-specific questionnaire, which we developed based on patient surveys prior to the start of the study.

Furthermore, patients had to fill out questionnaires assessing their social environment (Lubben social network scale), their physical disability (Modified Rankin Scale), their cognitive function (Trail-making-test), their intelligence (MWT-B), and their physical and mental wellbeing (EORTC QLQ-C30 and EORTC QLQ-INFO25) prior to surgery and at their follow-up appointment.

The patients were questioned in person until day 4 after surgery and during their follow-up appointment 6–8 weeks after surgery. The remaining questionnaires were given to the patients to fill in at home independently. Patients were reminded to fill out the questionnaires 3–4 weeks after surgery via telephone. Testing time was not timed in the patient group. However, a control group consisting of 10 individuals was timed for filling out all questionnaires and tests of the last visit.

### Study-specific questionnaire (SSQ)

We designed a study-specific questionnaire of only 19 items (Supplemental Material 1) as a short screening tool for depression that could be implemented in a routine clinical setting. This questionnaire was established based on existing depression screening tools and modified after feedback from patients.

Using Cronbach’s alpha, correlations with BDI-II, and CES-D as well as clinical expertise and experience, this questionnaire was transformed into a score-based questionnaire with a maximum of 49 points (Supplemental Material 2) at the end of the study.

Prior to transformation, the questions of the initial SSQ were correlated to the results of BDI, PHQ-9, and CES-D.

Because the SSQ was not a validated screening tool, no defined intervals were given between interviews using the SSQ. Therefore, the SSQ was used in a probative manner within shorter time intervals than predefined questionnaires (13 points in time, close intervals after histopathological diagnosis). These time intervals were included in the analysis regarding the SSQ.

### Data analysis

IBM SPSS (USA), Microsoft Excel, and SAS, Release 9.4, were used for statistical analysis. Patients were divided into three groups: patients with malignant primary brain tumors, patients with benign/low-grade primary brain tumors, and patients with metastatic cancer to the brain. For extended analysis, the patients were then divided into two subgroups: patients with benign brain lesions and patients with malignant tumors (metastases and high-grade glioma).

For quantitative analysis, we used mean values, standard error of the mean (SEM), and standard deviation (SD). Variance analysis was used to test for significant change of values over time separately for each patient group as described previously. If results showed significant changes, the Scheffé-test was added. For further analyses including all patients with malignant brain lesions including GBM and metastatic disease, we used ANOVA and Dunnett tests.

We compared the patients’ tumor diagnosis with their performance in CES-D, PHQ-9, Beck Depression Inventory, study-specific questionnaire, Lubben Social Network Scale, EORTC QLQ-C30, and EORTC QLQ-INFO25. Data was tested for correlations between the Lubben Social Network Scale and CES-D, the number of errors in MWT-B and CES-D, sex and CES-D, Modified Rankin Scale and CES-D as well as age and CES-D. For this purpose, we used the Pearson correlation coefficients.

*P*-values below 0.05 were considered significant.

Results > 16 points on CES-D were considered suggestive of depression.

If case numbers were low (specifically for CES-D), chi^2^ and Fisher’s tests were used for frequencies and distributions.

## Results

### General

**Fig. 1:**
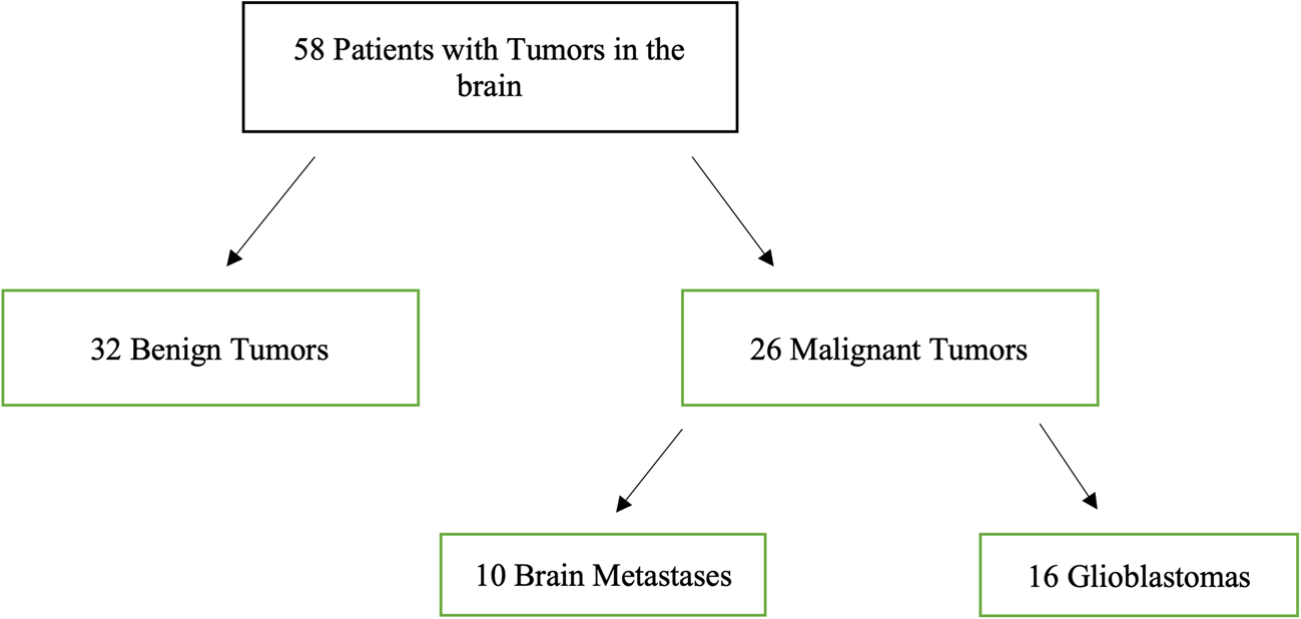
Distribution of tumor entities within the trial

Figure 1 illustrates the distribution of benign intracranial lesions and malignant brain tumors. Benign lesions included meningiomas, low-grade gliomas, pituitary tumors, and cavernous hemangiomas. Malignant brain tumors only included glioblastomas and brain metastases.

Three lesions were not able to be defined histologically or were identified as inflammatory processes. These patients were excluded from statistical analysis.

Tumor locations in patients with GBM were temporal, temporoinsular, frontal, occipital, parietooccipital, parietal, frontotemporal, temporobasal, basal, and multifocal on a single hemisphere as well as on both hemispheres. Isolated temporal tumors were the most frequent (3 patients).

We recruited 61 patients for this study; their ages ranged from 22 and 78 (57.2 ± 13.81) years. Fifty-seven percent were female, and 43% were male. Three patients were excluded after pathological diagnosis failed to show a tumor.

In the healthy control group, filling out all questionnaires and tests at the last follow-up appointment took between 10 min and 20 s and 20 min with a mean time of 14 min.

Only 32 patients of the 61 patients initially included (52%) completed the study. Not all of those 32 patients completed all questionnaires at each interview. The 29 patients that discontinued the study did so for the following reasons: impairment (10/29), missed follow-up (16/29), and secondary exclusion if histological workup did not diagnose a tumor (3/29).

Only 50% of the patients with GBM and 40% of patients diagnosed with brain metastasis completed all 6 interviews of the study, whereas 69% of the patients with benign lesions were able to complete all questionnaires until the final follow-up. For further analysis, we then stratified our patient cohort into two groups and combined patients with GBM and brain metastasis into one group of patients diagnosed with a malignant brain tumor compared to patients diagnosed with benign brain tumors.

Only one patient showed a positive screening test for social isolation on Lubben social network scale.

No correlation could be identified between sex and depression, intelligence scores and depression, as well as elevated risk for social isolation and depression.

There was a significant positive correlation between scores on CES-D and physical disability on MRS (preoperative correlation: 0.331, *p* = 0.015; follow-up correlation: 0.483, *p* = 0.013).

### Established depression scores

Prior to surgery and therefore before a diagnosis was made and communicated to the patient, scores on depression scales were similar for patients with malignant and benign lesions. CES-D, Beck Depression Inventory, and PHQ-9 showed positive correlation before surgery (CES-D and PHQ-9: 0.762 (*p* < 0.001), CES-D and BDI-II: 0.774 (*p* < 0.001), PHQ-9 and BDI-II: 0.758 (*p* < 0.001)).

Prior to surgery, the mean CES-D score in patients with benign tumors was 12.9 (± 10.5) points with 11/32 (34%) patients having a score of 16 points or more suggesting clinically relevant depression. The mean score in patients with malignant brain tumors was 12.3 (± 8.2) with the score adding up to 16 or more points in 9/25 patients (36%), respectively.

**Fig. 2:**
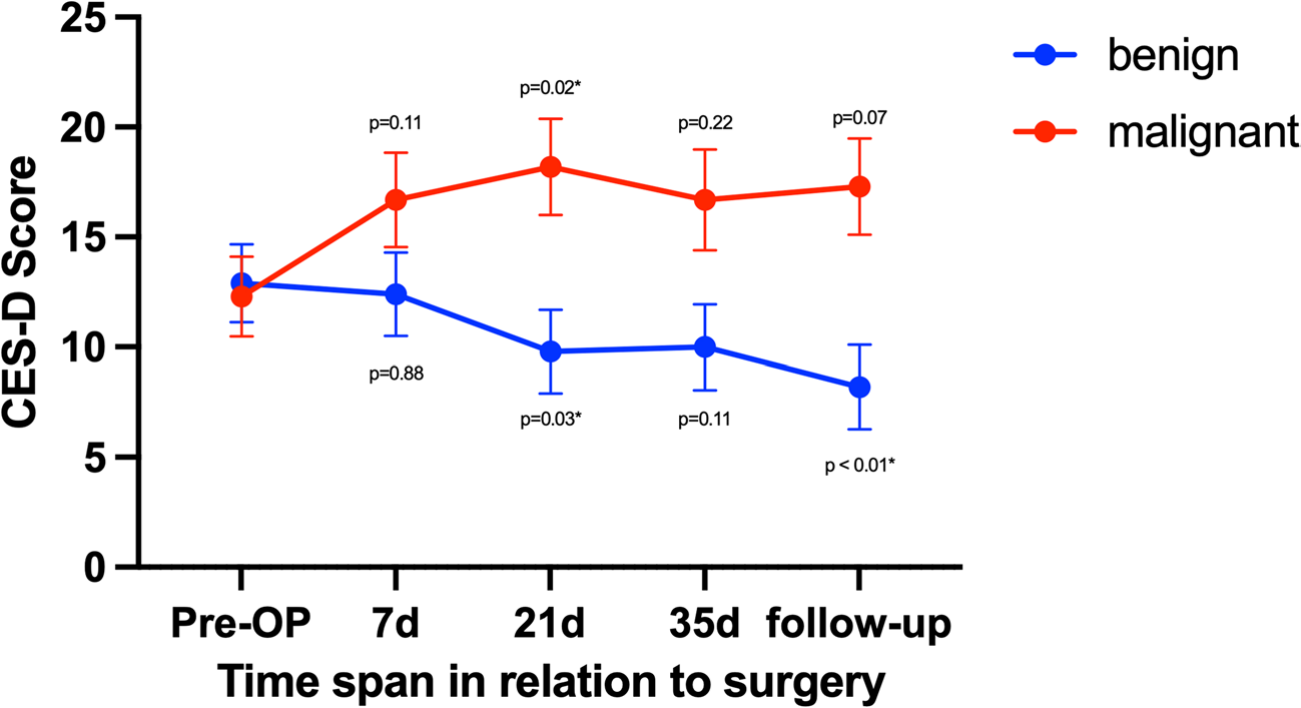
Comparison between patients with benign and malignant lesions with *p*-values < 0.05 marked with * via Dunnett tests on CES-D. Error bars show standard error of the mean (SEM). Decrease in scores for patients with benign tumors could be shown via one-way ANOVA with *p* = 0.049*. Increase in scoring for patients with malignant brain tumors could be shown after one-way ANOVA with *p* = 0.002*

Figure 2 shows that CES-D scores increased significantly for patients with malignant brain tumors (*p* = 0.029) and decreased significantly for patients with benign brain tumors (*p* = 0.049) over time. Furthermore, it could be shown that CES-D scores deviated significantly (*p* < 0.001) over time in a direct comparison between patients with benign and malignant brain tumors.

**Fig. 3:**
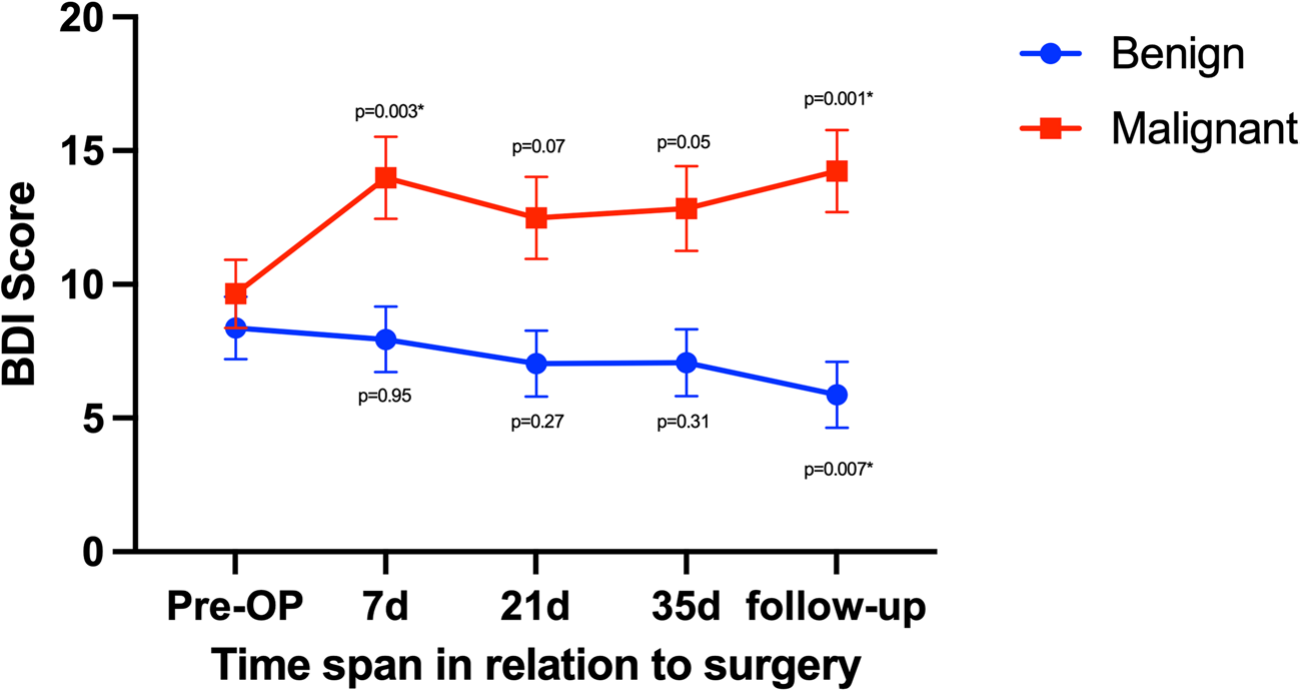
Comparison between patients with benign and malignant lesions with *p*-values < 0.05 marked with * via Dunnett tests on BDI. Error bars show standard error of the mean (SEM). Decrease in scores could be shown for benign brain tumors via one-way ANOVA with *p* = 0.02* and increase in scores could be shown for malignant brain tumors via one-way ANOVA with *p* = 0.002*

Similar results were seen for BDI tests, as presented in Fig. 3. The interaction between the patient group with benign tumors and the patient group with malignant tumors showed deviation with *p* < 0.0001.

When comparing patients with benign tumors to patients with GBM (metastases excluded), no significant trend towards increases or decreases in the depression scales could be shown for GBM alone. However, 21 days after surgery, 78.5% of patients with GBM showed pathological results (score of 16 points or more) on CES-D compared to 22% of patients with benign brain lesions, while 75% of patients with GBM showed pathological results on CES-D at follow-up, compared to 18% of patients with benign brain lesions (*p* = 0.002 and *p* = 0.007, respectively) (Table 1).

**Table 1:** Number of patients with CES-D scores of 16 points or more

Time of testing	Benign (%)	GBM (%)	Test	*p*-value
Pre-OP	11 (34%**)	5 (31%**)	Chi^2^	0.829
7 days post-OP	5 (22%**)	5 (56%**)	Fisher	0.096
21 days post-OP	5 (22%**)	7 (88%**)	Fisher	0.002*
35 days post-OP	5 (25%**)	4 (67%**)	Fisher	0.138
Follow-up	4 (18%)	6 (75%)	Fisher	0.007*

Values with *p* < 0.05 marked with *; ** = rounded to full percent. *Post-OP*, post-operation

In this study, adherence was low (52%). Reasons for low adherence differed between the patient groups and between different points in time. Patients with benign tumors mostly stated that they did not find time to fill out the high number of questionnaires during days 7 through 21 at home as well as during their follow-up appointment. Patients who had glioblastoma or brain metastases, however, did not complete the questionnaires due to physical or mental impairment and subsequently missed or changed their follow-up appointment. If patients with malignant brain tumors attended their follow-up appointment, all questionnaires were filled out.

### Study-specific questionnaire (SSQ)

Using the SSQ, there was a non-significant differential trend of patients diagnosed with GBM for the following items: patients diagnosed with GBM tended towards a higher level of sadness were more afraid to lose control, had less plans for the future, felt more powerless, had more changes in their social life, and described themselves as less emotionally stable when compared to patients with benign lesions or brain metastases.

None of the changes, however, were significant.

**Fig. 4:**
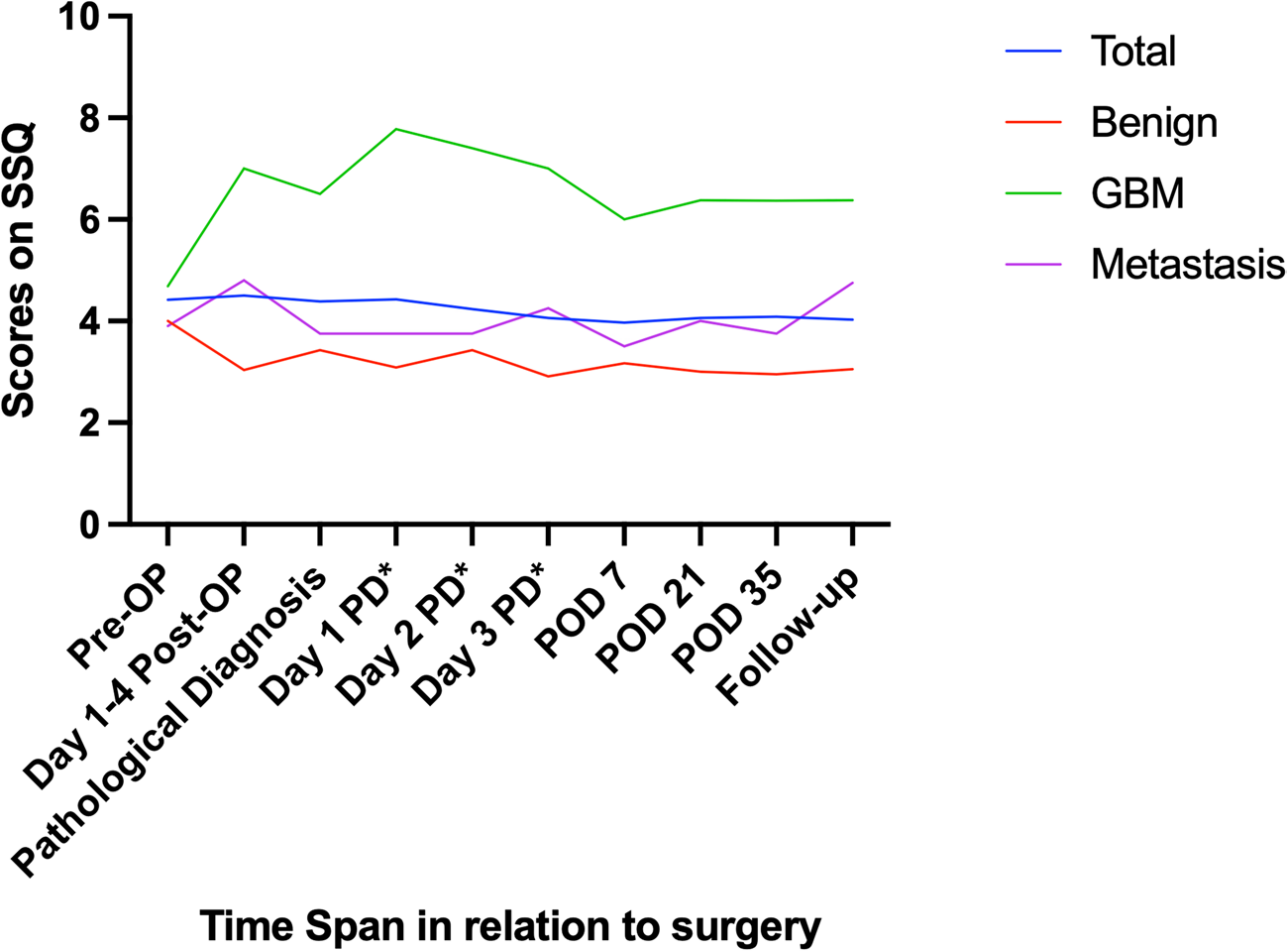
Scale from 0 to 10, 0 being feeling of having control, 10 being feeling of having lost control. PD* post histopathological diagnosis, POD postoperative day

However, the results showed tendencies towards differing results in patients with metastases and patients with GBM (as shown exemplarily in Fig. 4).

To validate, whether certain questions on the SSQ were suitable for detecting depression, we focused on correlation coefficients between the single items of the SSQ (Supplemental Material 1) and CES-D scoring results. We found correlations for the following questions on the questionnaire: 1, 2, 4, 7, 9, 11, 13, 14, 17, and 19 (*p* < 0.05).

As an example, patients who strongly agreed with the statement “I feel helpless” were more likely to score higher points in CES-D (correlation coefficient 0.477; *p* = 0.0003). Patients who gave an answer closer to “yes” when asked if they could cope with their diagnosis were much less likely to score high on CES-D (correlation coefficient − 0.581; *p* < 0.0001).

In some cases, correlations and weak correlations could be identified between CES-D and the study-specific questionnaire (SSQ).

A representative trend within the SSQ is illustrated in Fig. 4.

In addition to correlation with CES-D and BDI-II, the study-specific questionnaire was also tested for internal consistency using Cronbach’s alpha. Again, questions 1, 2, 4, 7, 9, 10, 11, 13, 14, 17, and 19 were included in the analysis. Questions 2, 4, and 13 matched least with the other questions (Table 2).

**Table 2:** Cronbach’s alpha standardized variables tested as internal consistency

	SSQ #1	SSQ #2	SSQ #4	SSQ #7	SSQ #9	SSQ #10	SSQ #11	SSQ #13	SSQ #14	SSQ #17	SSQ #19
Cronbach’s alpha	0.27	0.04	− 0.05	0.14	0.28	0.28	0.18	0.09	0.3	0.16	0.23

Deriving from these results, a new SSQ was designed using those questions that showed internal consistency and correlation to preexisting depression scales (Supplementary Material 2).

### Number needed to screen

Exemplarily, assuming a sensitivity of 85% for depression screening using CES-D [11] and a prevalence of 90% for patients with malignant brain tumors [2], a number needed to screen for detection of depression in patients with GBM of 1.59 with a false positive screening rate of 2.8%. was calculated (Table 3).

**Table 3:** Cross table based on 1000 patients with a prevalence of 90% for depression

	Detected by questionnaires				Detected by family physician	
	With depression	Without depression			With depression	Without depression
Positive testing	765	28	793	Positive testing	135	0
Negative testing	135	72	207	Negative testing	765	100
	900	100	1000		900	100

This table is relevant for those patients with worrisome screening results that are not identified by their family physician. This applies for 630 of 1000 patients, concluding in a number needed to screen of 1.59 (reciprocal of 630/1000).

## Discussion

The prevalence of depression in our patient cohort (up to 87.5% in patients with GBM) is similar to depression rates in GBM patients previously reported by Litofsky et al. [2]. Others report lower rates of 20–50% in patients with similar diagnoses [2, 12, 13]. Surprisingly, the discrepancy between the patients qualified as depressed based on established screening tools such as CES-D or BDI compared to those being diagnosed as depressed by their family physician was remarkable. Litofsky et al. showed that approximately 15% of patients are diagnosed with a clinically relevant depression by the family physician [2].

These numbers have been confirmed by our data, showing that 75% of our patients with glioblastoma needed further assessment for depression when followed up and 87.5% of patients showed scoring > 16 points on CES-D 21 days after surgery.

Overall, a clear dependency between the CES-D scoring and the tumors’ nature could be proven (*p* = 0.0063). Patients with malignant brain tumors (metastases and GBM) were more likely to develop symptoms of depression than patients with benign brain lesions.

Assuming that only 15% of patients with malignant brain tumors are diagnosed with depression by their family physician, 76.5% of depressions in patients with malignant brain tumors are not diagnosed and therefore not treated (0.15 × 0.9; 13.5% of brain tumor patients overall diagnosed correctly with a prevalence of 90%) (see Table 3).

This underscores not only the need for an increased awareness of all health care physicians but also the need for reliable and short depression screening tools for patients diagnosed with malignant brain tumors, e.g., GBM.

Knowing that depression is associated with a reduced mean overall survival [14], it seems crucial to diagnose and treat depression in patients who already have a very limited prognosis.

In consonance with this knowledge, a study by Mainio et al. also suspects depression to be a strong negative predictor for survival in patients with gliomas [15].

Any chronic disease is a known risk factor for depression [3, 16] and has previously been reported to increase the risk of depression by 45% [17].

Furthermore, substantial evidence shows a clear correlation between depression and quality of life [8–10, 18].

Based on our findings, we therefore strongly encourage routine screening for depression in patients with malignant brain tumors. We support this suggestion with the very low number needed to screen of 1.59.

For screening purpose, we adjusted the SSQ (Attachment 2) and are planning to further adapt the SSQ through application and then validation during the patients’ follow-up appointment. We plan to then adapt the SSQ again after testing of reliability, feasibility, evaluation of sensitivity, and evaluation of specificity. For screening purpose, we currently recommend additionally using established depression scales until the SSQ is fully established.

Our data showed first clear results 21 days after surgery with little change during the following weeks. We therefore suggest the time slot for screening to be around 3 weeks after surgery. Screening may be performed in combination with radiotherapy, for example. We emphasize that we could show that brain tumor patients only develop symptoms of depression after surgery, even though patients are already aware of their probable diagnosis prior to surgery through imaging. Screening, therefore, is not expedient prior to surgery.

Until a tumor-specific questionnaire is fully developed as a screening score system, we suggest the use of established screening questionnaires such as CES-D for screening.

If worrisome results are attained during screening, we suggest early integration in psychological support teams to improve quality of life and possibly overall survival on the long term.

## Limitations of the study

The patient number included in this trial is small. Although significant results could be obtained, a larger patient cohort might show clearer results.

Furthermore, we suspect a selection bias. Patients not able to complete questionnaires or other testing due to impairment were not included in the study. Also, patients with positive screening in BDI or CES-D testing were offered psycho-oncological support, possibly biasing later results in depression inventories.

Patients not receiving surgical therapy were excluded from the trial.

## Supplementary Information

Below is the link to the electronic supplementary material.

Supplementary file1 (PDF 106 KB)

Supplementary file2 (PDF 82 KB)

## Data Availability

Data and materials can be obtained from the Neurosurgical Department of the Medical Faculty Mannheim.

## Abbreviations

BDI-II: Beck Depression Inventory II
CES-D: Center for Epidemiologic Studies Depression Scale
CT: Computed tomography
EORTC QLQ C30: Organization for Research and Treatment of Cancer Quality of Life Questionnaire C30
EORTC QLQ-INFO25: Organization for Research and Treatment of Cancer Quality of Life Questionnaire INFO25
GBM: Glioblastoma
HPD: Histopathological diagnosis
MRI: Magnetic resonance tomography
MRS: Modified Rankin Scale
MWT-B: Mehrfach-Wortschatz-Intelligenztest 2 (German for: multiple vocabulary Intelligence Test B)
WHO: World Health Organization
CI: Confidence interval
IL-1: Interleukin 1
IL-6: Interleukin 6
IL-8: Interleukin 8
PHQ-9: Public Health Questionnaire 9
POD: Postoperative day
SD: Standard deviation
SEM: Standard error of the mean
SSQ: Study-Specific questionnaire
TNF-α: Tumor necrosis factor-α
