# Supplementary material for: Depression in brain tumor patients—early detection and screening
Source: Support Care Cancer. 2023 May 16;31(6):339. doi: 10.1007/s00520-023-07785-5 (PMC10188424; doi:10.1007/s00520-023-07785-5)
Supplement: Supplementary file 1 — Supplementary file1 (PDF 106 KB) [file 520_2023_7785_MOESM1_ESM.pdf]

Study-Specific Questionnaire – English Version

Please answer each Question with one mark per Question.

Example: ○—○—○—X—○

1. How much are you feeling restricted regarding quality of life?

Not at all ○—○—○—○—○ Very much

2. How positive is your mood?

Not at all positive ○—○—○—○—○ Very positive

3. How much faith do you have?

No faith at all ○—○—○ very much faith

4. How many changes in your social life have there been since your diagnosis?

Few changes ○—○—○ Many changes

5. Do you feel powerless?

Not at all ○—○—○ Very much

6. How often do you speak about your diagnosis to friends and family?

Never ○—○—○—○—○ Very often

7. How emotionally stable are you feeling?

Not stable at all ○—○—○—○—○ Very stable

8. In your eyes, how would you describe your current life situation?

Very bad ◯—◯—◯—◯—◯ Very good

9. How often do you think about the tumor?

Never ◯—◯—◯—◯—◯ Constantly

10. How well can you arrange yourself with your diagnosis?

Not at all ◯—◯—◯—◯—◯ Very well

11. Are you scared of losing control?

Not at all ◯—◯—◯—◯—◯ Very
