# Supplementary material for: Depression in brain tumor patients—early detection and screening
Source: Support Care Cancer. 2023 May 16;31(6):339. doi: 10.1007/s00520-023-07785-5 (PMC10188424; doi:10.1007/s00520-023-07785-5)
Supplement: Supplementary file 2 — Supplementary file2 (PDF 82 KB) [file 520_2023_7785_MOESM2_ESM.pdf]

Study-Specific Questionnaire – English Version

Please answer each Question with one mark per Question.

Example: O—O—O—X—O

1. How much are you feeling restricted regarding quality of life?

Not at all O—O—O—O—O—O—O—O—O—O Very much

2. How positive is your mood?

Not at all positive O—O—O—O—O—O—O—O—O—O Very positive

3. How sad are you feeling?

Not at all sad O—O—O—O—O—O—O—O—O—O Very sad

4. How much faith do you have?

No faith at all O—O—O—O—O—O—O—O—O—O very much faith

5. Do you have plans for the future?

Please choose:

- O None
- O Few
- O Some
- O Many

6. To what do you connect the tumor to?

Please Choose one option:

- O Foreign Body
- O Part of your body
- O Tumor
- O Temporary disruptive factor
- O Other: _________________

7. How many changes in your social life have there been since your diagnosis?

Please Choose:

- O A very large amount of changes
- O Many changes
- O Some changes
- O Few changes
- O No changes

8. When would you rather be informed about your diagnosis?

Please Choose:

- O Before the weekend
- O After the weekend
- O No preference

Reasons for choice (optional): ____
____
____
____

9. Do you feel powerless?

Not at all O—O—O—O—O—O—O—O—O—O Very much

10.How often do you speak about your diagnosis to friends and family?

Please Choose:

- O Never
- O Occasionally
- O Sometimes
- O Often

11.How emotionally stable are you feeling?

Not stable at all O—O—O—O—O—O—O—O—O—O Very stable

12. How much zest of life are you currently experiencing?

None at all O—O—O—O—O—O—O—O—O—O A large amount

13.In your eyes, how would you describe your current life situation?

Please Choose:

- O Bad
- O Mostly bad
- O Mostly good
- O Good

14.How often do you think about the tumor?

Please Choose:

- O Never
- O Around once a week
- O Multiple times per week
- O Every day
- O Multiple times a day
- O Constantly

15. How much improvement are you expecting from the surgery?

None at all O—O—O—O—O—O—O—O—O—O Maximum

16.How happy are you with the care at the hospital?

Not at all O—O—O—O—O—O—O—O—O—O Extremely

17.How well can you cope with your diagnosis?

Not at all O—O—O—O—O—O—O—O—O—O Very well

18. Are you joining in activities that give you joy?

Please choose:

- O Never
- O Sometimes
- O Often
- O Very often

19.Are you scared of losing control?

Not at all O—O—O—O—O—O—O—O—O—O Very
